# Pilot Study of a Multilevel Mobile Health App for Substance Use, Sexual Risk Behaviors, and Testing for Sexually Transmitted Infections and HIV Among Youth: Randomized Controlled Trial

**DOI:** 10.2196/16251

**Published:** 2020-03-17

**Authors:** David Cordova, Jaime Munoz-Velazquez, Frania Mendoza Lua, Kathryn Fessler, Sydni Warner, Jorge Delva, Nicole Adelman, Youth Leadership Council, Angela Fernandez, Jose Bauermeister

**Affiliations:** 1 School of Social Work University of Michigan Ann Arbor, MI United States; 2 School of Social Service Administration University of Chicago Chicago, IL United States; 3 Corner Health Center Ypsilanti, MI United States; 4 School of Social Work Boston University Boston, MA United States; 5 See Acknowledgments; 6 School of Nursing University of Pennsylvania Philadelphia, PA United States

**Keywords:** youth, mHealth, illicit drugs, sex behavior, HIV, primary care

## Abstract

**Background:**

Preventing and reducing substance use disorders, sexually transmitted infections (STIs)/HIV, and teen pregnancy, and the associated risk behaviors (ie, substance use and sexual risk behaviors) among youth remain public health priorities in the United States. Equally important is improving the uptake of STI/HIV testing among the youth. Mobile health (mHealth) apps may be a solution to ameliorate these public health concerns; however, few mHealth preventive interventions have demonstrated efficacy in reducing substance use or sexual risk behaviors or improving the uptake of STI/HIV testing among the youth, particularly in clinic settings.

**Objective:**

This small-scale study aimed to examine the feasibility of conducting a pilot randomized controlled trial (RCT). We evaluated the effects of Storytelling 4 Empowerment (S4E), relative to enhanced usual practice, on the potential mechanisms by which behavior change occurs, namely clinician-youth risk communication, prevention knowledge, and substance use and sexual risk refusal self-efficacy. We also assessed the ability to measure targeted outcomes of past 30-day substance use (ie, alcohol, tobacco, and other drug use), condomless sex, and alcohol or drug use before sex, as well as the uptake of STI/HIV testing.

**Methods:**

Employing community-based participatory research principles, 50 youths aged 13 to 21 years were recruited from a youth-centered community health clinic in Southeast Michigan, randomized sequentially to either S4E or enhanced usual practice, and assessed at baseline, immediately postintervention, and 30 days postintervention. S4E consists of 3 modules, including alcohol and drug use, tobacco, and STI/HIV.

**Results:**

Relative to youth in the enhanced usual practice group, S4E participants demonstrated higher youth-clinician risk communication (mean 3.22, SD 1.67) and increases in prevention knowledge (∆ score mean 0.36, SD 0.51) and self-efficacy (∆ score mean 0.16, SD 0.47). In addition, youth in the S4E group showed reductions in the proportions of past 30-day overall substance use (Cohen *h*=0.71, 95% CI 0.15 to 1.27), as well as past 30-day alcohol (Cohen *h*=0.71, 95% CI 0.15 to 1.27), tobacco (Cohen *h*=0.17, 95% CI −0.39 to 0.73), and drug use (Cohen *h*=1.28, 95% CI 0.72 to 1.84). The results also suggest a reduction in the proportion of youths who reported past 30-day condomless sex (Cohen *h*=0.18, 95% CI −0.38 to 0.74) and alcohol use before sex (Cohen *h*=0.44, 95% CI −0.12 to 1.00). Finally, the findings also demonstrated an increase in the proportion of youths who reported STI/HIV testing over time (Cohen *h*=0.16, 95% CI −0.39 to 0.72).

**Conclusions:**

The findings suggest the feasibility of a small-scale pilot RCT. S4E demonstrated shifts in the hypothesized direction, reducing substance use, sexual risk behaviors, and improving the uptake of STI/HIV testing among youth in a clinic setting. The findings suggest that a larger RCT may be warranted.

**Trial Registration:**

ClinicalTrails.gov NCT03855410, https://clinicaltrials.gov/ct2/show/NCT03855410.

## Introduction

### The Prevalence of Sexually Transmitted Infections/HIV and Associated Risk Behaviors Among the Youth

Sexually transmitted infections (STIs), including HIV, and teen pregnancy, remain significant public health concerns among youth in the United States [1-4]. STIs and HIV infection have been linked to infertility, cancer, and increasing vulnerability to opportunistic infections [5,6]. In addition, teen pregnancy has been linked to low income, poverty, and low educational attainment [7]. Therefore, preventing and reducing STIs, HIV, and teen pregnancy, as well as associated risk behaviors such as substance use and sexual risk behaviors, remain critical public health priorities.

Substance use and sexual risk behaviors may directly or indirectly increase the risk of STIs, HIV, and teen pregnancy. These behaviors often increase during adolescence, highlighting the need to intervene in these behaviors throughout this developmental period of increased vulnerability [8-10]. National epidemiologic data suggest that substance use behaviors, including alcohol, tobacco, and other drug use, are widespread among the youth [11]. Substance use behaviors often parallel other risk behaviors [12] and have been linked to increased sexual risk behaviors among the youth [8,13,14]. In addition to preventing and reducing substance use and sexual risk behaviors, STI and HIV testing are key strategies to reduce the high rates of STI/HIV infection among the youth [15,16]. Despite the Centers for Disease Control and Prevention guidelines, many youths are not routinely screened for STIs [17], and 90.7% of 9th- to 12th-grade students report having never been tested for HIV [11]. Therefore, there remains an urgent need to identify settings and tools that may be leveraged to improve the uptake of STI/HIV testing among youth.

### Leveraging Youth-Centered Community Health Clinics and Mobile Health Apps to Prevent Sexually Transmitted Infections/HIV Among the Youth

Youth-centered community health clinics may be an ideal setting to engage youth in prevention services. Evidence supports that compared with adult-focused clinics and AIDS service organizations, youth are more likely to seek substance use and sexual risk prevention and risk reduction services from youth-centered community health clinics [18,19]. However, relatively few interventions have been developed and tested in clinic settings [20,21]. Leveraging clinic settings, in combination with technology, may have great utility in identifying substance use, sexual risk behaviors, and STI/HIV testing solutions for youth.

Mobile health (mHealth) refers to medical or public health initiatives and practices supported by mobile devices such as tablets and the internet [22]. Among a limited yet growing body of research, mHealth interventions have been pilot-tested and demonstrated positive shifts in reducing substance use, sexual risk behaviors, or increasing STI/HIV testing among youth [23-28]. For example, researchers have shown that brief mHealth interventions reduce marijuana use among youths aged 15 to 24 years at 3 months postintervention [24] and heavy alcohol consumption among young bisexual men at 3 months postintervention [26]. Other research has shown that brief mHealth interventions can improve the uptake of HIV [23,25,28,29] and STI testing [23] and decrease the frequency of condomless sex [25].

### Limitations of Scientific Knowledge on Mobile Health Preventive Interventions

Although scientific advancements on mHealth preventive interventions have been made, several important limitations exist. First, few mHealth preventive interventions focus on substance use and concurrent risk behaviors (ie, sexual risk behaviors) in younger adolescents (aged <18 years) [25,30], missing the opportunity to affect a key developmental period of enhanced risk-taking [31]. Second, interventions targeting sexual risk behaviors and uptake of STI/HIV testing have focused primarily on young men who have sex with men [23,25,29], with few interventions focused on other vulnerable populations. Indeed, stark HIV disparities among men who have sex with men exist, accounting for 87% of new HIV diagnoses among youths aged 13 to 24 years [32]. Also important are racial and ethnic minority youth and adolescent women who constitute additional vulnerable populations of youth [32-34]. Third, to date, interventions have focused primarily on linking the youth to STI/HIV testing sites [23,29], with relatively few preventive interventions focused on the youth once they arrive at the clinic. Although drawing youth to the clinic is an important first step, it does little good if effective prevention services are not provided once the youth arrive at the clinic. Simply focusing on drawing the youth to clinics may create missed opportunities for engaging the youth in additional prevention strategies, particularly among those who are unaware of their engagement in risky behaviors [30]. To address these limitations, we conducted a small-scale randomized controlled trial (RCT) to pilot-test the feasibility of a multilevel mHealth preventive intervention among a diverse sample of youth in a clinic setting.

### The Storytelling 4 Empowerment Mobile Health Preventive Intervention App

Employing community-based participatory research (CBPR) principles [35], we developed Storytelling 4 Empowerment (S4E) [30,36]. Guided by ecodevelopmental [37] and empowerment theories [38], S4E aims to reduce substance use, sexual risk behaviors, and improve uptake of STI/HIV testing through improving clinician-youth risk communication, prevention knowledge, and self-efficacy [30]. This is accomplished through a multilevel mHealth app that provides interactive, targeted, and tailored content focused on the prevention of substance use and sexual risk behaviors. This mHealth app is then followed up with a clinician-initiated prevention and risk reduction face-to-face encounter, providing clinicians the opportunity to reinforce content provided to youth during their interaction with the mHealth app. Because S4E has been shown to have a positive user experience, an effective user interface, and high feasibility and acceptability among both youth and clinicians [30,39], a next important step is to conduct a small-scale pilot RCT to determine the feasibility of S4E and examine shifts in potential mechanism of change and the ability to measure substance use, sexual risk behaviors, and uptake of STI/HIV testing among a diverse sample of youth. We believe our S4E multilevel approach may offer advantages over other approaches for several reasons. First, our intervention was developed with and for the targeted community. For example, youth helped steer the development of S4E with regard to the user experience and user interface [36]. Researchers affirm that community-engaged research employing CBPR principles may lead to enhanced uptake of, and optimally efficacious, preventive interventions [35]. Second, our intervention is developmentally and culturally congruent, utilizing spaces and tools that align with youth perspectives. Specifically, youth-centered clinics are safe spaces for many youths, thereby providing a potentially high-impact context to improve public health. Furthermore, approximately 95% of the youth report having access to mobile devices [40], which may be leveraged to deliver risk behavior solutions to this vulnerable population. Finally, our S4E approach was grounded in decades of science and informed by prevention principles [41]. For example, S4E is theory driven, targets multiple levels, and focuses on multiple risk behaviors that often co-occur [30,36,39].

### Purpose of the Study

The purpose of this study was to conduct a small-scale pilot RCT to determine the feasibility of S4E, relative to enhanced usual practice. We evaluated changes in the potential mechanisms of change, namely clinician-youth risk communication, prevention knowledge, and self-efficacy over time. We also assessed the ability to measure substance use, sexual risk behaviors, and uptake of STI/HIV testing over time among a diverse sample of youth in a clinic setting. Given the small-scale pilot nature of our study and sample size, statistical significance was deemphasized. Rather, our goal was to assess feasibility and establish the critical parameters necessary to inform a larger future RCT.

## Methods

### Participants

We recruited youth and clinicians between October 2016 and July 2017 from a youth-centered community health clinic located in Southeast Michigan that offers a full range of health care, mental health, and supportive services to young people as they transition to adulthood.

**Figure 1:**
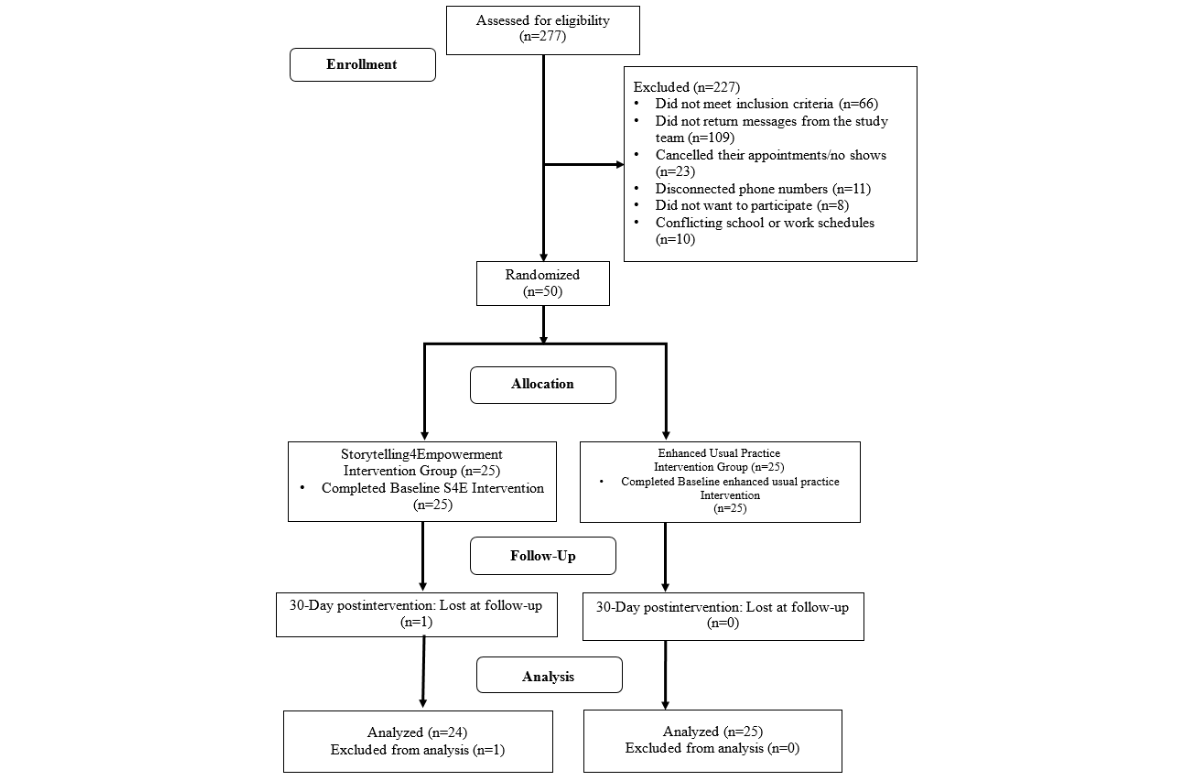
Consolidated Standards of Reporting Trials table.

Youth recruitment occurred during the clinic’s health appointment reminder phone calls. To be eligible for this study, youth had to (1) be aged between 13 and 21 years, (2) live in Southeast Michigan without plans to move out of the area during the study period, (3) have a scheduled appointment with a participating clinician, and (4) report no prior history of psychiatric hospitalization. Of the 277 youths who were screened for eligibility, 211 met the inclusion criteria. Of these, 109 youths did not return calls or messages left by the study team, 23 canceled or did not show for their scheduled health appointment, 11 had disconnected or incorrect phone numbers, 10 reported conflicting schedules with school or work, and 8 refused to participate. Therefore, of the 211 eligible youths, 50 were successfully recruited (see Figure 1).

Clinician recruitment occurred during weekly clinic staff meetings, and all clinicians at the health clinic were eligible to participate if they (1) worked in Southeast Michigan and (2) worked with our target population. The study team approached 8 clinicians, of which 7 agreed to participate. Both youths and clinicians who expressed interest in participating in the study were contacted by the study team to screen for eligibility, to enroll, and to complete study consent protocols. To protect the confidentiality of the youth aged between 13 and 17 years, a waiver of parental permission was obtained. This waiver was in accordance with the state of Michigan’s Title X Program and Public Health Code, MCL 333.6121, which states that a minor aged 17 years or younger can consent to sexual and reproductive health and substance use services without parental knowledge. Both participating youths older than 18 years and clinicians were presented with consent through a comprehensive written waiver of documentation that did not require a signature from the participant or legally authorized representative while containing all the elements of informed consent required by the Health and Human Services’ regulations and policy.

### Sample Characteristics

In line with other research pilot testing mHealth preventive interventions [24,26], the sample size for this study was 50, with 25 youth participants randomized to the S4E group and 25 randomized to the enhanced usual practice group. Of the 50 youths, 41 (82%) identified as female, followed by 4 (8%) males, and 4 (8%) transmales, and 1 refused to respond. The mean age of the youths was 18.82 years (SD 2.1, range 13-21). The racial composition of these 50 youths consisted of 23 (46%) non-Hispanic white, 21 (42%) black, 1 (2%) Native American, 4 (8%) ascribing to more than one race, and 1 (2%) selecting Other. Regarding the youths’ educational attainment, 36% (18/50) of youths reported having completed some college, whereas 30% (15/50) reported having completed high school. The remaining 34% (17/50) of youths reported having completed a grade between 7th and 11th.

Among the 7 clinicians who agreed to participate, 6 (86%) identified as female, with a mean age of 43.14 years (SD 7.95, range 34-56), and 5 (71%) were non-Hispanic white, followed by 1 (14%) Hispanic or Latino and 1 (14%) Asian. They reported an average of 10.86 years (SD 7.45, range 1-22) of medical practice in their respective specialties: 71% (5/7) practiced family medicine and 29% (2/7) were pediatricians. Finally, 71% (5/7) of the clinicians reported having lived in the area where they work for more than 10 years, and 29% (2/7) reported having lived in the area for fewer than 10 years.

### Study Design

This study employed community-based participatory research principles [35]. A youth leadership council, clinic director, and staff were involved in all aspects of this research, including preparing and submitting the proposal to fund this study, identifying the target population, developing the study design, and disseminating the study findings (eg, publications). This study consisted of a 2 (group) × 2 (time) small-scale pilot RCT. Youth participants were randomly assigned to either the S4E experimental group or enhanced usual practice control group via sequential randomization [42]. Data were collected on tablets and captured using Research Electronic Data Capture, a Health Insurance Portability and Accountability Act–compliant, web-based app that is hosted on secure servers at the University of Michigan Medical School. To reduce potential bias, eligible participants completed health surveys that included questions regarding substance use, sexual risk behaviors, and STI/HIV testing practices, before randomization. All youths arrived 1 hour before their scheduled health care appointment to have the study explained to them in detail, to provide consent, and to complete baseline assessments in a reserved room, all of which took approximately 30 min. Youths participated in the intervention (S4E or enhanced usual practice) in a reserved room with internet connection for approximately 30 min, while they waited for their health appointment. Participants completed the S4E intervention on tablets provided to them by the research team. The S4E mHealth version tested in this study was developed for Apple’s operating system (iOS). Because this was a phase I/II pilot study, we had participants complete the S4E intervention in the clinic to have a more controlled environment. Youth participants were assessed at baseline before their health appointment, immediately postintervention, and 30 days postbaseline. Clinicians were assessed at baseline and immediately postintervention for each health appointment.

We retained 49 participants at our 30-day follow-up (49/50, 98% retention rate). Youth participants received a total of US $60 in incentives, corresponding to US $20 at baseline and US $40 at the 30-day follow-up. Through a collaborative process, the clinic and research team decided to provide a US $2000 incentive to benefit the entire community health clinic for providing us with meeting space and for the clinicians’ time on the project, rather than give individual incentives to the participating clinicians.

### Study Groups

#### Storytelling 4 Empowerment Group

**Figure 2:**
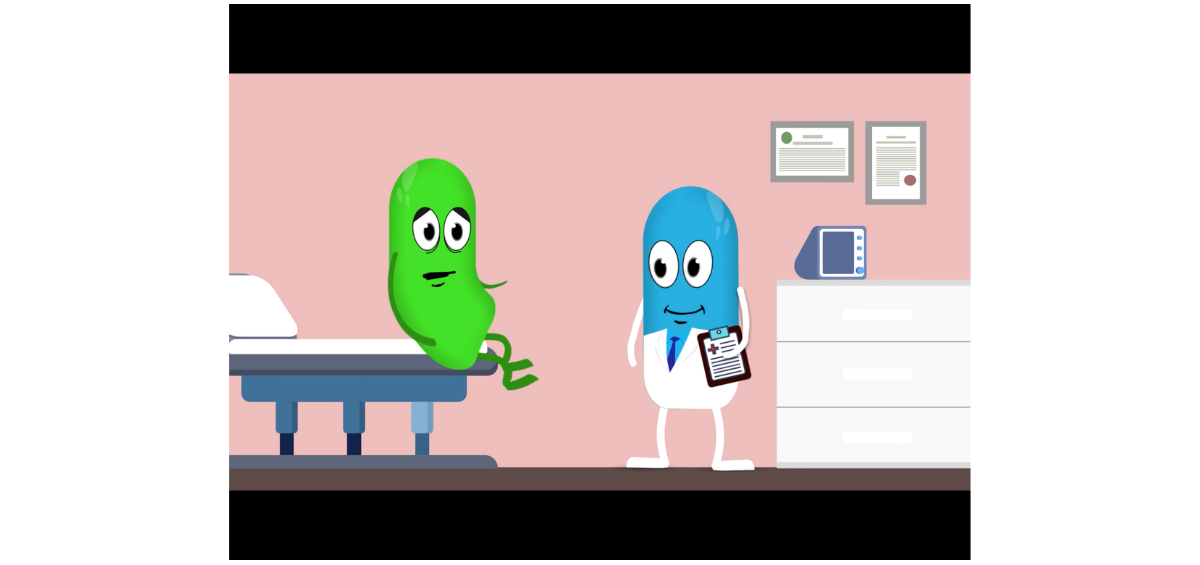
Storytelling 4 Empowerment animated storytelling scenario.

**Figure 3:**
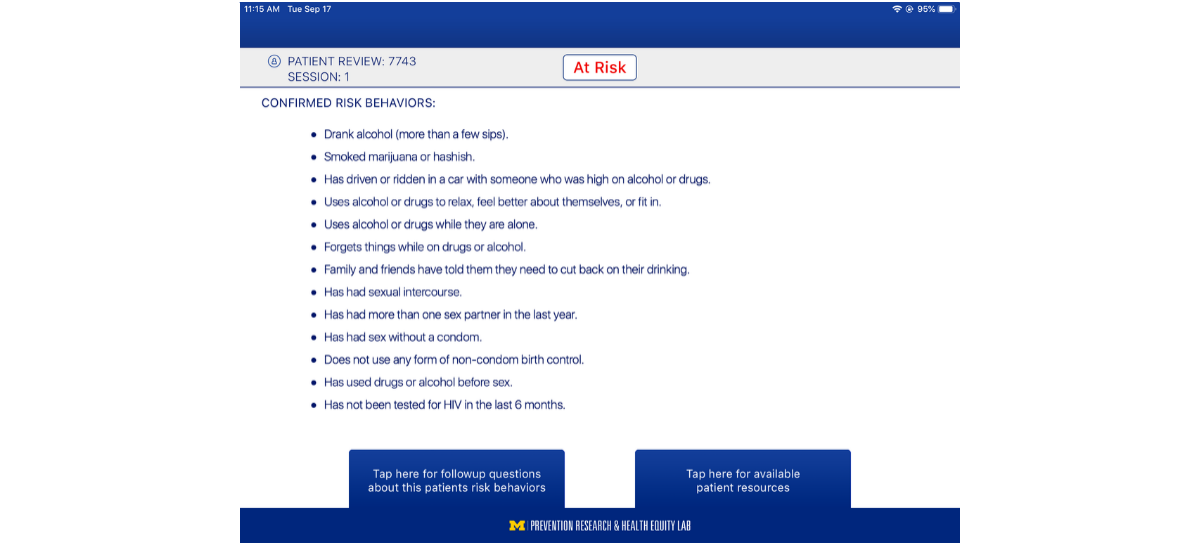
Clinician-facing Storytelling 4 Empowerment application highlighting youth risk behaviors.

The S4E intervention content was generated through community-university research involving youth-led groups in conjunction with scientific prevention principles [30]. Theory-driven, S4E takes a multilevel approach and is guided by an ecodevelopmental [37] and empowerment framework [38]. Youths in the S4E group received targeted, tailored prevention content based on their responses to the S4E risk behavior assessment, which includes the Car, Relax, Alone, Forget, Friends, Trouble screener [43]. This assessment is intended to identify the youths’ specific risk behaviors based on the past year and lifetime reports of substance use, sexual risk behaviors, and past 6-month STI/HIV testing practices (Multimedia Appendix 1). From an empowerment perspective, the scores prompt risk-specific interactive prevention content (eg, short animated storytelling scenarios, interactive diagram of body health activities) for the S4E youth (Figure 2). This interactive content is delivered via 3 modules (ie, alcohol and drug use, tobacco, and STI/HIV) and aims to improve prevention knowledge, self-efficacy, and refusal skills while linking youth to important adult figures. From an ecodevelopmental perspective, the clinician-facing app contained the participants’ health appointment information, their assigned group, and their S4E risk assessment responses. Both the youth and clinician S4E apps work synergistically, providing clinicians access to the youths’ risk responses, motivational interviewing scripts to facilitate clinician-youth communication, and resources to local services that are based on the youths’ risk behaviors (Figure 3). Overall, the S4E intervention aims to improve substance use and sexual risk prevention knowledge, self-efficacy, and refusal skills, as well as to facilitate clinician-youth risk communication. By focusing on these malleable factors, the overarching goal of the S4E intervention is disease prevention (ie, substance use disorders, STI/HIV) and health promotion (ie, STI/HIV testing) through reductions in substance use, sexual risk behaviors, and improvements in STI/HIV testing among youth.

### Enhanced Usual Practice Group

The participating youth-centered community health clinic’s usual practice consists of primary care, mental health, sexual and reproductive health, substance use prevention, support, and education (eg, Women, Infants, and Children Supplemental Food Program), and gender-affirming health care to youths aged 12 to 25 years. Participants in both the S4E group and enhanced usual practice received the clinic’s usual services. In addition, participants in the enhanced usual practice group received a binder with a printed PDF version of the S4E tobacco module content to view before their health care appointment. The materials consisted of epidemiologic statistics and prevention health information related to tobacco and cigarette use. The Youth Leadership Council strongly recommended an enhanced usual practice control group so that all youth participants in the trial would receive some form of additional prevention services.

### Measures

#### Demographics

Youth and clinicians completed a demographic survey that asked their age, ethnicity, race, and gender identity. In addition, youth reported educational attainment, and clinicians reported their medical specialty, years of clinical experience, and length of time residing in the area where they practiced.

#### Clinician-Youth Communication (Immediately Postintervention)

Both clinicians (Cronbach alpha=.81, 9-item) and youth (Cronbach alpha=.95, 9-item) interactions during the clinic visit were assessed via items extracted and adapted from the matched pair instrument (MPI) [44] immediately postintervention. These items assessed the process and content of the communication, including the language used and behaviors performed by clinicians related to substance use and sexual risk behaviors services. A sample statement for clinicians is as follows: *Encouraged the patient to express his or her thoughts concerning drug use and/or sexual risk behaviors.* A sample statement for youths is as follows: *My doctor encouraged me to express my thoughts concerning drug use and/or sexual risk behaviors.* Both clinicians and youth responded to items in the measure using a 6-point Likert-type scale (0=*not applicable*, 5=*strongly agree*). To make the MPI sum scores more interpretable, the scores were rescaled to their original 6-point metric by dividing the sum total by the total number of items before significance testing.

#### Self-Efficacy Outcomes

##### Substance Use Refusal Skills

Youths’ substance use refusal skills were assessed through 2 separate items on a 4-point scale (1=*very hard* to 4=*not very hard*). Sample questions included the following: *Pretend your best friend offered you a drink of beer or wine and you did not want it. How hard would it be to say no?* and *Pretend your best friend offered you some marijuana and you did not want it. How hard would it be to say no?*

##### Sexual Risk Behavior Refusal Skills

One item was used to assess youths’ sexual risk behavior refusal skills. The statement read the following: *I can say no to sex if my partner and I do not have a condom.* Responses were on a 5-point agreement scale (1=*strongly disagree*, 5=*strongly agree*).

##### Prevention Knowledge Outcomes

###### Substance Use Prevention Knowledge

Youths’ knowledge about alcohol or drugs and tobacco products was assessed through 2 separate items. Items included the following: *If I use alcohol or drugs, I will have more health problems than other people* and *If I use tobacco products, I will have more health problems.* Responses were on a 4-point agreement scale (1=*disagree a lot* to 4=*agree a lot*).

###### Sexual Risk Prevention Knowledge

Sexual risk prevention knowledge was measured through 2 separate items related to the effectiveness of condom use. Items included the following: *Condoms help prevent pregnancy,* and *If I have sex without a condom, I am likely to get HIV/STIs.* Responses were on a 5-point agreement scale (1=*strongly disagree,* to 5=*strongly agree*).

##### Behavioral Outcomes

Substance use behaviors, sexual risk behaviors, and HIV/STI testing were dichotomized for analysis, whereby *0* was used for *No* (ie, no risk present), and *1* indicated *Yes* (ie, risk was present) for the item in question.

##### Substance Use Behaviors (Baseline and 30-Day Postbaseline)

Youths’ lifetime and past 30-day substance use behaviors were assessed using items adapted from the Monitoring the Future study [45,46]. Sample items included the following: *Have you ever had any beer, wine, wine cooler, or liquor to drink*? and *Have you had more than a few sips of alcohol on more than one occasion during the past 30 days?*

##### Sexual Risk Behaviors (Baseline and 30-Day Postbaseline)

Participants’ lifetime and past 30-day sexual risk behaviors were assessed using items extracted from the Sexual Behavior Instrument [47]. Sample items include *Have you ever had vaginal, anal, or oral sex without using a condom?* and *In the past 30 days, about how often have you had vaginal, anal, or oral sex without using a condom?*

##### Sexually Transmitted Infection and HIV Testing (Baseline, Immediately Postintervention, 30-Day Postbaseline)

We assessed youths’ lifetime and most recent STI and HIV testing. Example questions included the following: *Have you ever been tested for HIV?* and *Did you receive an STI test?*

### Analytic Strategy

Given the modest sample size and goals of a pilot RCT, significance testing by group was deemphasized [23]. Rather, we determined whether outcomes shifted in the hypothesized direction and gathered the necessary parameters to use in a larger RCT in the future [48]. The data analytic strategy consisted of 4 steps. First, we conducted a descriptive statistical analysis on demographic and outcome variables at baseline and used chi-square tests and analysis of variance to test for significant group differences at baseline. Second, we conducted chi-square tests and 2-tailed *t* tests to determine if there were significant differences by group in attrition and elapsed time between baseline and 30-day follow-up assessments. Third, we determined group differences in change of potential mechanisms of change (ie, self-efficacy, prevention knowledge, clinician-youth risk communication) over time. Finally, we assessed the ability to measure between-group differences (ie, S4E vs control) in the change of reported substance use, sexual risk behaviors, and STI/HIV testing over time. We also assessed the ability to measure within-group differences in outcomes among participants in the S4E intervention group. Differences were determined using proportion change (∆ %) for categorical outcomes and mean change (∆ score, absolute net gains) over time for continuous outcomes. We report the observed effect sizes for the outcome change difference scores by group using Cohen *d* for continuous outcomes and Cohen *h* for binary outcomes, which measure the difference between 2 proportions (*h*=2arcsin × (sqrt *P*_1_) − 2arcsin × (sqrt *P*_2_), where *P*_1_=proportion 1 and *P*_2_=proportion 2). Effect sizes were estimated as small (*d/h*<.20), medium (.20≥*d/h*≤.45), and large (*d/h*>.45) to observe the magnitude of differences [49]. All analyses were performed in SPSS version 24 [50], with the exception of Cohen *h* power calculations, which were performed in R’s version 3.5.2 PWR package [51].

## Results

### Comparability of Groups

As shown in Table 1, chi-square tests and analyses of variance results suggest no significant S4E vs control group differences at baseline on any demographic characteristic (eg, race), lifetime or past 30-day substance use, sexual risk behaviors, and lifetime STI/HIV testing. The absence of significant differences in these variables at baseline suggests that our trial’s randomization procedures were successful. The median number of days between baseline and follow-up was 31 (mean 32.63, SD 8.62); no significant differences by group in the number of days between baseline and 30-day follow-up assessments were observed using a 2-tailed *t* test, t_46.36_=0.42, *P*=.68. In addition, no significant differences in attrition by group were observed, χ^2^_1_=0.0, *P*=.92.

**Table 1:** Baseline comparisons by group on demographic and behavioral outcomes (N=50).

Outcomes		S4E^a^ (n=25)	Control (n=25)	*t* test/chi-square (*df*)	*P* value
Age (years), mean (SD)		18.6 (2.15)	19.0 (2.19)	−0.58 (47.98)^b^	.56
**Gender, n (%)**					
	Female	21 (84)	20 (80)	1.0 (2)	.61
	Male	2 (8)	2 (8)	1.0 (2)	.61
	Transmale	1 (4)	3 (12)	1.0 (2)	.61
	Transfemale	—^c^	—	1.0 (2)	.61
	Refuse to answer	1 (4)	—	1.0 (2)	.61
**Race, n (%)**					
	Black	11 (44)	10 (40)	2.4 (4)	.66
	White	10 (40)	13 (52)	2.4 (4)	.66
	Native American	1 (4)	—	2.4 (4)	.66
	More than one race	2 (8)	2 (8)	2.4 (4)	.66
	Other	1 (4)	—	2.4 (4)	.66
**Lifetime substance use, n (%)**					
	Lifetime alcohol use	21 (84)	21 (84)	—	—
	Lifetime tobacco use	16 (64)	17 (68)	0.9 (1)	.77
	Lifetime other drug use	14 (56)	13 (52)	0.1 (1)	.78
**Past 30-day substance use, n (%)**					
	Past 30-day substance use (ATOD^d^)	15 (60)	16 (64)	0.1 (1)	.77
	Past 30-day alcohol use	10 (40)	14 (56)	1.3 (1)	.26
	Past 30-day tobacco use	7 (28)	11 (44)	1.4 (1)	.23
	Past 30-day other drug use	12 (48)	7 (28)	1.3 (1)	.26
**Lifetime sexual risk behaviors, n (%)**					
	Lifetime condomless sex	20 (80)	20 (80)	—	—
	Lifetime alcohol use before sex	9 (36)	14 (56)	2.0 (1)	.16
	Life drug use before sex	7 (28)	8 (32)	0.2 (1)	.69
**Past 30-day sexual risk behaviors, n (%)**					
	Past 30-day condomless sex^e^	13 (62)	13 (62)	0.2 (1)	.67
	Past 30-day alcohol use before sex^e^	2 (10)	3 (14)	0.2 (1)	.67
	Past 30-day drug use before sex^e^	3 (14)	4 (19)	0.1 (1)	.73
	Lifetime HIV/STI^f^ testing	22 (88)	20 (80)	0.6 (1)	.44

^a^S4E: Storytelling 4 Empowerment.

^b^*t* test for age; rest are chi-square values.

^c^Data are not applicable.

^d^ATOD: alcohol, tobacco, other drug use.

^e^Among sexually active youth.

^f^STI: sexually transmitted infection.

### Intervention Effects on Clinician-Youth Risk Communication

Immediately postintervention, youths in the S4E group reported higher levels of clinician-youth risk communication (mean 3.22, SD 1.67), relative to the youths in the control group (mean 2.96, SD 1.63; t_46.77_=0.56; *P*=.58). Although these group differences were not statistically significant, the estimated effect size (Cohen *d*=0.16, 95% CI −0.41 to 0.72) yielded a small effect size. Similarly, S4E clinicians reported higher levels of clinician-youth risk communication (mean 3.47, SD 1.13), relative to clinicians in the control group immediately postintervention (mean 3.23, SD 1.02; t_45.98_=0.75; *P*=.45). Although these differences were not statistically significant, the estimated effect size (Cohen *d*=0.22, 95% CI −0.34 to 0.79) yielded a small value.

### Intervention Effects on Self-Efficacy

As shown in Table 2, youths in the S4E group reported greater change scores in substance use self-efficacy alcohol refusal (∆ score mean 0.22, SD 0.67), relative to the youths in the control group (∆ score mean 0.16, SD 0.55; t_42.79_=0.32; *P*=.75; Cohen *d*=0.10) and for drug refusal (∆ score mean 0.09, SD 0.68), relative to the youths in the control group (∆ score mean 0.08, SD 0.70; t_44.51_=0.05; *P*=.96; Cohen *d*=0.01). Yet, both the S4E group (∆ score mean 0.08, SD 0.78) and the control group (∆ score mean 0.13, SD 0.92) showed an increase in sex self-efficacy (t_44.06_=−0.19; *P*=.85; Cohen *d*=−0.06). In contrast to the between-group effects, intervention effects within the S4E group (Table 3) for both substance use items, alcohol refusal (∆ score mean 0.21; t_22_=1.55, *P*=.14; Cohen *d*=0.38) and drug use refusal (∆ score mean 0.09; t_21_=0.62; *P*=.54; Cohen *d*=0.12), yielded small to medium effect sizes. Similarly, sexual risk self-efficacy change scores within the S4E group (∆ score mean 0.08; t_23_=0.53; *P*=.60; Cohen *d*=0.10) yielded small effect sizes.

### Intervention Effects on Prevention Knowledge

As shown in Table 2, youths in the S4E group reported higher overall gains in substance use prevention knowledge for tobacco use (∆ score mean 0.30, SD 0.77), relative to the youths in the control group (∆ score mean 0.16, SD 0.80; t_45.93_=0.64; *P*=.53; Cohen *d*=0.18). The S4E group reported similar gains of prevention knowledge for alcohol or drug use (∆ score mean 0.35, SD 0.65), relative to the youths in the control group (∆ score mean 0.20, SD 1.00; t_41.46=_0.61; *P*=.54; Cohen *d*=0.18). In addition, S4E youths reported overall gains for sexual risk prevention knowledge (pregnancy prevention, ∆ score mean 0.08, SD 0.65; STI/HIV prevention, ∆ score mean 0.25, SD 1.22), relative to the control group (pregnancy prevention, ∆ score mean 0.00, SD 0.41; t_38.29_=0.53; *P*=.60; Cohen *d*=0.15 and STI/HIV prevention, ∆ score mean −0.17, SD 1.74; t_43.35_=0.96; *P*=.34; Cohen *d*=0.28). Both outcomes yielded small to medium effect sizes. In contrast to the between-group effects, intervention effects within the S4E group (Table 3) for both substance use prevention knowledge (tobacco use ∆ score mean 0.34; t_22_=2.58; *P*=.02; Cohen *d*=0.49 and alcohol or drug use ∆ score mean 0.31; t_22_=1.91; *P*=.07; Cohen *d*=0.50) and the sexual risk prevention knowledge-pregnancy prevention (∆ score mean 0.08; t_23_=0.62; *P*=.54; Cohen *d*=0.10) and STI/HIV prevention (∆ score mean 0.25; t_23_=1.00; *P*=.32; Cohen *d*=0.21) yielded small to medium effect sizes.

**Table 2:** Self-efficacy and prevention knowledge by group.

Outcomes		S4E^a^, ∆ score, mean (SD)	Control, ∆ score, mean (SD)	Independent *t* test (*df*)	Cohen’s *d* (S4E vs control)	Cohen *d* 95% CI
**Substance use self-efficacy**						
	Alcohol refusal	0.22 (0.67)	0.16 (0.55)	0.32 (42.79)	0.10	−0.47 to 0.66
	Drug refusal	0.09 (0.68)	0.08 (0.70)	0.05 (44.51)	0.01	−0.56 to 0.59
**Sexual risk self-efficacy**						
	Condomless sex	0.08 (0.78)	0.13 (0.92)	−0.19 (44.06)	−0.06	−0.61 to 0.50
**Substance use prevention knowledge**						
	Use of tobacco products	0.30 (0.77)	0.16 (0.80)	0.64 (45.93)	0.18	−0.40 to 0.76
	Use of alcohol or drugs	0.35 (0.65)	0.20 (1.00)	0.61 (41.46)	0.18	−0.39 to 0.74
**Sexual risk prevention knowledge**						
	Pregnancy prevention	0.08 (0.65)	0.00 (0.41)	0.53 (38.29)	0.15	−0.41 to 0.71
	STI^b^/HIV prevention	0.25 (1.22)	−0.17 (1.74)	0.96 (41.35)	0.28	−0.28 to 0.84

^a^S4E: Storytelling 4 Empowerment.

^b^STI: sexually transmitted infection.

**Table 3:** Storytelling 4 Empowerment intervention effects on self-efficacy and prevention knowledge (n=25).

Outcomes		S4E^a^ baseline, mean (SD)	S4E follow-up, mean, (SD)	∆ score mean	Paired *t* test (*df*)	Cohen *d*	Cohen *d* 95% CI
**Substance use self-efficacy**							
	Alcohol refusal	3.57 (0.59)	3.78 (0.52)	0.21	1.55 (22)	0.38	−0.94 to 0.19
	Drug refusal	3.50 (0.86)	3.59 (0.67)	0.09	0.62 (21)	0.12	−0.68 to 0.44
**Sexual risk self-efficacy**							
	Condomless sex	3.25 (0.85)	3.33 (0.76)	0.08	0.53 (23)	0.10	−0.66 to 0.46
**Substance use prevention knowledge**							
	Use of tobacco products	3.09 (0.79)	3.43 (0.59)	0.34	2.58 (22)	0.49	−0.10 to 1.07
	Use of alcohol or drugs	3.30 (0.56)	3.61 (0.58)	0.31	1.91 (22)	0.50	−0.07 to 1.07
**Sexual risk prevention knowledge**							
	Pregnancy prevention	3.50 (0.72)	3.58 (0.50)	0.08	0.62 (23)	0.10	−0.43 to 0.69
	STI^b^/HIV prevention	2.67 (1.24)	2.92 (1.10)	0.25	1.00 (23)	0.21	−0.35 to 0.77

^a^S4E: Storytelling 4 Empowerment.

^b^STI: sexually transmitted infection.

### Between-Group Intervention Effects on Substance Use Behaviors

Overall, participant reports of substance use at baseline were not significantly different (Table 1). However, the S4E group reported a greater reduction in any substance use (ie, ATOD) relative to the control group (3/25, 12% vs 0/25, 0%; Table 4). Although chi-square testing (χ^2^_2_=4.5; *P*=.10) suggests that these proportion differences between groups were marginally significant, the estimated proportion change effect size difference between groups (Cohen *h*=0.71) yielded a large effect size. We then deconstructed past 30-day substance use into past 30-day alcohol, tobacco, and other drug use to determine between-group intervention effects on each of these outcomes.

**Table 4:** Past 30-day behavior outcome proportion change scores by group.

Outcomes		∆ S4E^a^	∆ Control	Cohen *h* (S4E vs control)	Cohen *h,* 95% CI
**Substance use, n (%)**					
	Substance use (ATOD^b^)	3 (−12)	0 (0)	0.71	0.15 to 1.27
	Alcohol use	3 (−12)	0 (0)	0.71	0.15 to 1.27
	Tobacco use	2 (−8)	1 (−4)	0.17	−0.39 to 0.73
	Other drug use	3 (−12)	2 (+8)	1.28	0.72 to 1.84
**Sexual risk behaviors, n (%)^c^**					
	Condomless sex	2 (−10)	1 (−5)	0.18	−0.38 to 0.74
	Alcohol use before sex	1 (− 5)	0 (0)	0.44	−0.12 to 1.00
	Drug use during sex	2 (−10)	2 (−10)	N/A^d^	N/A

^a^S4E: Storytelling 4 Empowerment.

^b^ATOD: alcohol, tobacco, other drug use.

^c^Sexual risk behaviors are based on responses from sexually active participants (n=42).

^d^Not applicable.

#### Alcohol Use

Overall, 84% (42/50) of participants reported lifetime alcohol use. Relative to participants in the control group, S4E group participants reported a greater reduction in past 30-day alcohol use at 30-day follow-up (3/25, 12% vs. 0/25, 0%). Although chi-square testing (χ^2^_2_=3.9; *P*=.14) suggests that this proportion change difference was not statistically significant between groups, the estimated proportion change effect size between groups (Cohen *h=*0.71) yielded a large effect size (Table 4).

#### Tobacco Use

Overall, 66% (33/50) of participants reported lifetime tobacco use. Participants in the S4E group reported a greater decrease in tobacco use, as compared with participants in the control group at 30-day follow-up (2/25, 8% vs 1/25, 4%). Although chi-square testing (χ^2^_1_=0.4; *P*=.50), suggests that these proportion differences were not statistically significant, the estimated effect size difference across groups (Cohen *h*=0.17) yielded a small effect size (Table 4).

#### Other Drug Use

Overall, 54% (27/50) of participants reported lifetime drug use. Participants in the S4E group reported a reduction (3/25, 12%) in drug use at 30-day follow-up, relative to an increase in drug use (2/25, 8%) among participants in the control group. Although chi-square testing (χ^2^_2_=2.9; *P*=.23) suggests that these proportion differences were not statistically significant, the estimated effect size difference across groups (Cohen *h* =1.28) yielded a large effect size (Table 4).

### Between- Group Intervention Effects on Sexual Risk Behaviors

#### Condomless Sex

Overall, 80% (40/50) of participants reported engaging in lifetime condomless sex. Relative to sexually active participants in the control group (n=21), sexually active S4E group participants (n=21) reported a greater reduction in past 30-day condomless sex at 30-day follow-up (2/21, 10% vs 1/21, 5%). Although chi-square testing (χ^2^_2_=0.2; *P*=.91) suggests that these proportion differences were not statistically significant, the estimated effect size difference between groups (Cohen *h*=0.18) yielded a small effect size (Table 4).

#### Alcohol Use Before Sex

Overall, 46% (23/50) of participants reported lifetime alcohol use before sex. Relative to sexually active participants in the control group (n=21) who reported no change, sexually active S4E group (n=21) participants reported a reduction in alcohol use before sex at 30-day follow-up (1/21, 5% vs 0/21, 0%). Although chi-square testing (χ^2^_2_=2.3; *P*=.32) suggests that these proportion differences were not statistically significant, the estimated effect size difference between groups (Cohen *h*=0.44) yielded a medium effect size (Table 4).

#### Drug Use Before Sex

Overall, 15 (30%) participants reported lifetime drug use before sex. Both the control and S4E group participants reported similar reductions in drug use before sex at 30-day follow-up (2/21, 10% vs 2/21, 10%; Table 4).

### Within-Group Intervention Effects on Substance Use Behaviors

As shown in Table 5, of the 25 youths in the S4E group, 12% (3/25) reported a decrease of any substance use from baseline assessment to 30-day follow-up (χ^2^_1_=10.9; *P*<.001; Cohen *h*=0.24). We then separated past 30-day substance use into past 30-day alcohol, tobacco, and other drug use to determine within-group intervention effects on each of these outcomes. S4E intervention effects for alcohol use from baseline assessment to 30-day follow-up showed a 12% (3/25) decrease in alcohol use (χ^2^_1_=3.6; *P*=.06; Cohen *h*=0.24). Similarly, 8% (2/25) of the S4E participants reported a decrease in tobacco use from baseline assessment to 30-day follow-up (χ^2^_1_=14.6; *P*<.001; Cohen *h*=0.19). In addition, 12% (3/25) of S4E youth reported a decrease in other drug use from baseline assessment to 30-day follow-up (χ^2^_1_=17.0; *P*<.001; Cohen *h*=0.24). Although chi-square significance was deemphasized, S4E intervention effects on substance use behaviors yielded small to medium effect sizes.

**Table 5:** Past 30-day Storytelling 4 Empowerment intervention effects on behaviors from baseline to follow-up (n=25).

Outcomes		S4E^a^ baseline, n (%)	S4E follow-up, n (%)		∆ score, n (%)		Cohen *h*		Cohen *h,* 95% CI	
**Substance use** **behavior**										
	Substance use (ATOD^b^)	15 (60)		12 (48)		3 (−12)		0.24		−0.32 to 0.80
	Alcohol use	10 (40)		7 (28)		3 (−12)		0.24		−0.31 to 0.81
	Tobacco use	7 (28)		5 (20)		2 (−8)		0.19		−0.37 to 0.74
	Other drug use	12 (48)		9 (36)		3 (−12)		0.24		−0.32 to 0.80
**Sexual risk behaviors^c^**										
	Condomless sex	13 (62)		11 (52)		2 (−10)		0.19		−0.37 to 0.75
	Alcohol use before sex	2 (10)		1 (5)		1 (−5)		0.18		−0.38 to 0.74
	Drug use before sex	3 (14)		1 (5)		2 (−10)		0.33		−0.23 to 0.89

^a^S4E: Storytelling 4 Empowerment.

^b^ATOD: alcohol, tobacco, other drug use.

^c^Sexual risk behaviors are based on responses from sexually active participants (n=21).

### Within-Group Intervention Effects on Sexual Risk Behaviors

As shown in Table 5, within-group intervention effects on S4E sexual risk behaviors from baseline assessment to 30-day follow-up had small to medium effect sizes that helped establish differences. Specifically, S4E reports of condomless sex decreased by 10% (2/21) from baseline assessment to 30-day follow-up (χ^2^_1_=2.9; *P*=.09; Cohen *h* =0.19). Moreover, decreases in alcohol use before sex (1/25, 5%; χ^2^_1_=8.97; *P*=.003; Cohen *h*=0.24) and drug use before sex (2/25, 10%; χ^2^_1_=8.47; *P*=.004; Cohen *h* =0.33) were observed.

### Intervention Effects on Sexually Transmitted Infections/HIV Testing

We sought to determine whether STI/HIV testing behaviors varied by group over time, independently of participants’ prior lifetime STI/HIV testing behaviors. At baseline, of the 50 youths, 42 (84%) reported having been tested for STI/HIV during their lifetime (Table 1). Moreover, no baseline lifetime STI/HIV testing differences were found by group. Relative to the control group, participants in the S4E group reported an overall higher uptake of STI/HIV testing across the trial (11/25, 44% vs 13/25, 52%). Although chi-square testing (χ^2^_1_=0.3; *P*=.57) suggests these differences were not statistically significant, the estimated effect size (Cohen *h*=0.16, 95% CI −0.39 to 0.72) yielded a small value.

## Discussion

### Principal Findings

The findings suggest the feasibility of a small-scale pilot RCT and demonstrated hypothesized shifts in reducing substance use, condomless sex, and alcohol use before sex, as well as improving uptake of STI/HIV testing among a diverse sample of youth. The estimated proportion change effect sizes of our behavioral outcomes (ie, substance use, sexual risk behaviors, STI/HIV testing) and potential mechanisms of change (ie, clinician-youth communication, prevention knowledge, self-efficacy) between the S4E and enhanced usual practice control groups yielded small to large effect sizes. Drawing on previous literature, the findings provide evidence for the promise of S4E in preventing and reducing substance use and sexual risk behaviors [23,27]. Reducing substance use and sexual risk behaviors and improving uptake of STI/HIV testing have been identified as key strategies to improve the health of young people in the United States [10,15,16]. Pilot testing S4E advances the scientific knowledge on mHealth preventive interventions and has important public health implications.

We demonstrated the feasibility of measuring intermediate outcomes. The potential mechanisms underlying the hypothesized shifts in substance use, sexual risk behaviors, and STI/HIV testing among youth in the S4E group may be partially explained by improvements in clinician-youth risk communication, substance use refusal self-efficacy, and prevention knowledge. Specifically, relative to youth in the control group, S4E participants demonstrated higher levels of clinician-youth communication immediately postintervention, as well as higher levels of substance use refusal self-efficacy and prevention knowledge at 30-day follow-up. The present design precludes us from formal mediation testing. However, these findings build on previous research indicating that clinician-youth communication, self-efficacy, and STI/HIV prevention knowledge may be pathways through which S4E has an effect on substance use, sexual risk behaviors, and STI/HIV testing [39]. Future research should include at least 3 time points to allow for formal mediation analysis [52], especially because few pathways by which mHealth preventive interventions affect behavioral outcomes have been identified [53].

We demonstrated the ability to measure outcomes targeted by S4E. Evaluation of the S4E intervention suggests reductions in overall licit and illicit substance use behaviors among youth. When we separated substance use behaviors, S4E helped decrease the proportion of youths who engaged in alcohol, tobacco, or other drug use at 30-day follow-up. These findings have important public health implications because reducing substance use behaviors among the youth aligns with the nation’s prevention goals and strategies to ameliorate substance use–related morbidity and mortality [15]. Furthermore, our findings are similar to those of other researcher’s pilot testing preventive interventions and lend support to the promise that mHealth strategies have in preventing and reducing youth risk behaviors [24-26].

The findings that S4E demonstrated hypothesized shifts in reducing condomless sex and alcohol use before sex have important public health implications because these risk behaviors are widespread among youth [11]. Therefore, the findings that S4E participants show reductions in the proportion of youth who engage in condomless sex or alcohol use before sex have important public health implications, as these risk behaviors are linked to increased vulnerability to STIs, HIV, and unplanned pregnancy—outcomes that disproportionately affect the youth [54]. Contrary to what we hypothesized, we did not find a between-group effect on drug use before sex; however, the findings suggest a statistically significant within-group change among S4E group participants. It may be that the 30-day follow-up time period is not sufficient in duration to capture the long-term effects of S4E relative to the control group on drug use before sex. Therefore, future research should examine the effects of S4E on drug use before sex over a longer period. Importantly, findings also suggest that S4E demonstrated shifts in improving uptake of STI/HIV testing among youth. This is especially important in the realm of public health, as improving STI and HIV testing uptake among youth is a key strategy to reducing the burden of STI and HIV infection among this vulnerable population [16,54,55]. Taken together, our small-scale pilot RCT suggests the feasibility of S4E, including determining intermediate outcomes, ability to measure behavioral outcomes, as well as demonstrated hypothesized shifts in reducing substance use, sexual risk behaviors, and STI/HIV testing among youth over 30 days, which aligns with other research pilot testing technology-based interventions on these behaviors [23-28]. It is also important to note that some researchers have found that technology-based interventions have a moderate health impact on exercise over 6 weeks [56]. Thus, in general, an important future research direction is to examine both the short and long-term effects of mHealth preventive interventions. In addition, this study focused on a sample of youths seeking care in a youth-centered community health clinic. Youths who are currently not in care may be more vulnerable to substance use and sexual risk behaviors, and future research could target this population.

Our findings have important future research implications and suggest that examining the efficacy of S4E in a larger RCT may be warranted. Implementation science designs might offer other alternatives to the traditional RCT, including stepped-wedge design and type 1 hybrid studies, which may increase the practicality of exploring intervention effects within real-life contexts [57,58]. Furthermore, given the multilevel approach of S4E, a sequential multiple assignment randomized trial (SMART) may lead to an optimally efficacious preventive strategy through the optimization of dose based on response (or lack thereof) to the intervention [59-62]. SMART is an innovative design that provides evidence for individualized decision making through adaptive interventions for the prevention of substance use and sexual risk behaviors [59,60]. In addition, our findings have important clinical implications. Our previous research establishing high feasibility and acceptability of S4E among both clinicians and youth [30,39] in conjunction with the findings of this pilot RCT endorses the implementation of strategies that support the youth-focused clinical health care workforce, especially if we are to achieve the nation’s public health goals laid out by initiatives such as the Substance Abuse and Mental Health Services Administration Strategic Plan for years 2019-2023 [63], National Prevention Strategy for increasing HIV/STI testing [64], ending the HIV Epidemic in the United States by 2030 [65], and teen pregnancy objectives in healthy people by 2020 [66].

### Study Limitations

Several limitations are worth noting. First, the sample in this study is not representative of the clinic population, limiting the generalizability of our findings. However, we demonstrated hypothesized shifts in prominent substance use, sexual risk behaviors, and STI/HIV testing in S4E youth, the majority of whom identified as racial and ethnic minorities. Second, the reliance on self-reported risk behavior outcomes is a limitation. Future research should consider access to medical charts as part of the study design. Third, our control group received a printed version of the S4E tobacco module content. Future research should use an attention- and time-matched control group design [67,68]. That is, an mHealth app focused on youth risk behaviors with similar intended dosage as S4E may be used in a future RCT to examine the differential effects of mHealth apps. Another limitation is that of the 211 potential participants eligible to participate in the trail; we could only reach 91 youth (43.1%). Of these, we successfully enrolled 50 youth (54.9%); however, these enrollment rates are similar to other research focused on mHealth preventive interventions with vulnerable populations [26]. The sample size of 50 is a study limitation; however, the sample size is akin to other research focused on pilot testing mHealth preventive interventions [24,26]. Finally, clinicians were not randomly assigned and provided usual practice to participants in both the experimental and control groups. Thus, there is potential for contamination between groups; however, contamination is highly unlikely given that the 2 groups are vastly different. Importantly, any potential bias would bias the findings toward the null, because clinicians could deliver S4E prevention strategies to youth in the control group.

### Conclusions

In summary, this study’s findings suggest the feasibility of a small-scale pilot RCT. S4E may have the potential as an mHealth strategy to reduce substance use and sexual risk–related outcomes such as STIs, HIV, and unplanned pregnancy among youth. In addition, the findings suggest that S4E demonstrated hypothesized shifts in improving uptake of STI/HIV testing, which is important for reducing the transmission and acquisition of STIs and HIV infection among youth. These findings advance scientific knowledge on mHealth preventive interventions and contribute toward improving public health through the identification of potential technology-based, youth substance use, and sexual risk behavior solutions.

## Appendix

Multimedia Appendix 1 S4E youth risk behavior assessment.

Multimedia Appendix 2 CONSORT-eHEALTH checklist (V 1.6.1).

## Abbreviations

ATOD: alcohol, tobacco, other drug use
CBPR: community-based participatory research
mHealth: mobile Health
MPI: Matched Pair Instrument
RCT: randomized controlled trial
S4E: Storytelling For Empowerment
SMART: Sequential Multiple Assignment Randomized Trial
STI: sexually transmitted infection
