# Supplementary figures and images for: Pilot Study of a Multilevel Mobile Health App for Substance Use, Sexual Risk Behaviors, and Testing for Sexually Transmitted Infections and HIV Among Youth: Randomized Controlled Trial
Source: JMIR Mhealth Uhealth. 2020 Mar 17;8(3):e16251. doi: 10.2196/16251 (PMC7109616; doi:10.2196/16251)

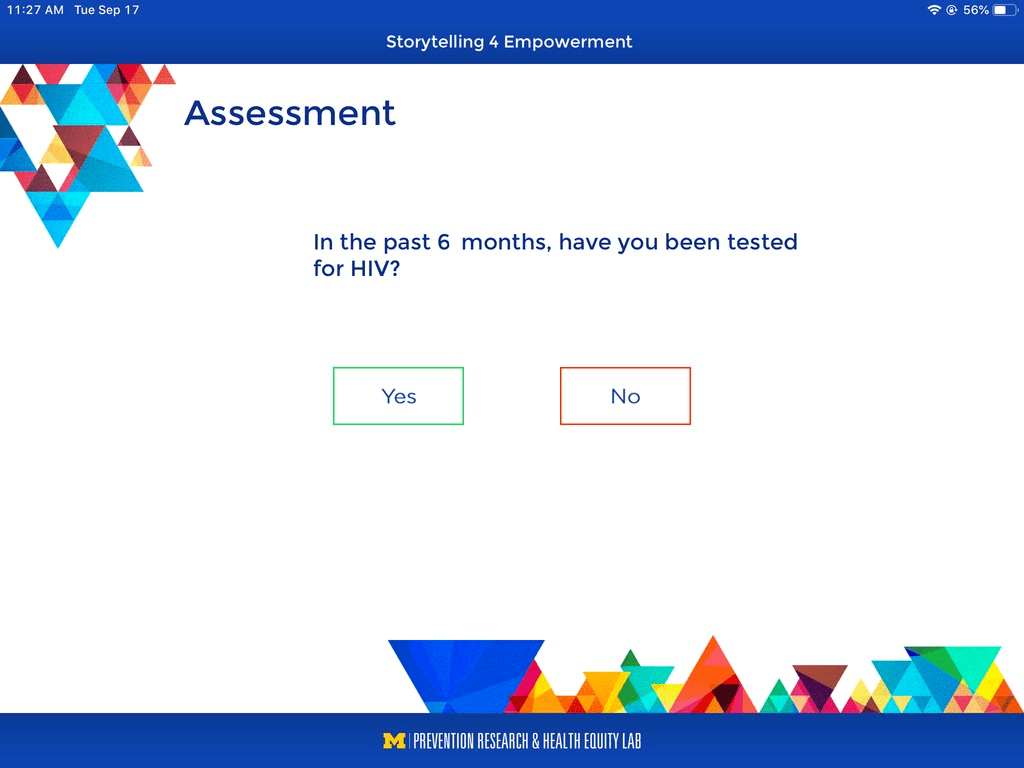

Supplement: Multimedia Appendix 1 [file mhealth_v8i3e16251_app1.png]
